# Over-the-air federated learning: Status quo, open challenges, and future directions

**DOI:** 10.1016/j.fmre.2024.01.011

**Published:** 2024-02-09

**Authors:** Bingnan Xiao, Xichen Yu, Wei Ni, Xin Wang, H. Vincent Poor

**Affiliations:** a Key Laboratory for Information Science of Electromagnetic Waves (MoE), Department of Communication Science and Engineering, Fudan University, Shanghai 200433, China; b Data61, Commonwealth Scientific and Industrial Research Organisation (CSIRO), Sydney, NSW 2122, Australia; c Department of Electrical and Computer Engineering, Princeton University, Princeton, NJ 08544, USA

**Keywords:** Machine learning, Federated learning, Over-the-air federated learning, Multiple-input multiple-out, Reconfigurable intelligent surface, Security, Privacy

## Abstract

The development of applications based on artificial intelligence and implemented over wireless networks is increasingly rapidly and is expected to grow dramatically in the future. The resulting demand for the aggregation of large amounts of data has caused serious communication bottlenecks in wireless networks and particularly at the network edge. Over-the-air federated learning (OTA-FL), leveraging the superposition feature of multi-access channels, enables users at the network edge to share spectrum resources and achieves efficient and low-latency global model aggregation. This paper provides a holistic review of progress in OTA-FL and points to potential future research directions. Specifically, we classify OTA-FL from the perspective of system settings, including single-antenna OTA-FL, multi-antenna OTA-FL, and OTA-FL with the aid of the emerging reconfigurable intelligent surface technology, and the contributions of existing works in these areas are summarized. Moreover, we discuss the trust, security and privacy aspects of OTA-FL, and highlight concerns arising from security and privacy. Finally, challenges and potential research directions are discussed to promote the future development of OTA-FL in terms of improving system performance, reliability, and trustworthiness. Specifical challenges to be addressed include model distortion under channel fading, the ineffective OTA aggregation of local models trained on substantially unbalanced data, and the limited accessibility and verifiability of individual local models.

## 1 Introduction

The envisioned sixth-generation (6G) of mobile communication systems has attracted significant attention in academic and industrial communities [1]. An important trend in discussions of 6G is a shifting of machine learning (ML) tasks from central cloud infrastructures to the network edge, capitalizing on the computational potential of edge devices and the flexibility of network connectivity [2], [3]. Federated learning (FL), a distributed learning framework, is particularly well-suited for edge applications [4], [5], [6], [7]. Initially proposed by McMahan et al. [8], FL has recently gained considerable traction. In FL settings, geographically distributed users train their own models using local data and then transmit their local model parameters or gradients to a base station (BS) for model aggregation. The BS subsequently returns the obtained global model to the users, repeating this process until model convergence [9]. Unlike traditional centralized learning settings, FL does not necessitate the transmission of large amounts of training data, thereby reducing communication costs and helping ensure data privacy to a significant extent [10].

### 1.1 Over-the-air federated learning

The concept of over-the-air (OTA) computation, also known as AirComp, was introduced by Nazer et al. [11] to leverage the signal superposition characteristics of wireless multiple access channels (MACs) for function computation. OTA computation offers the advantage of resource consumption reduction since the BS only needs to handle functions uploaded by users rather than individual data. Dedicated radio resource allocation for each user is unnecessary, making the OTA communication-computation approach highly suitable for the model aggregation process in FL.

**Fig. 1:**
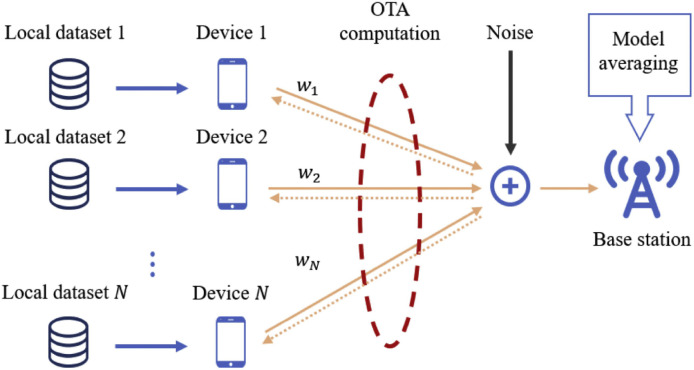
**An illustration of the system model of OTA-FL**. Users perform several local updates and then transmit their respective models updates through MAC. Upon the OTA aggregation, the updates are sent to the BS for the global model update.

Over-The-Air Federated Learning (OTA-FL) represents a revolutionary approach to wireless federated learning (WFL) in multi-access channels, capitalizing on the signal superposition capability of radio. As illustrated in Fig. 1, OTA-FL empowers users to transmit their individual model updates within the same spectrum, eliminating the necessity for a BS to allocate dedicated radio resources. Consequently, this approach circumvents the challenge of spectrum resource consumption increasing linearly with the rising number of participating users.

OTA-FL finds a multitude of practical applications across various scenarios. Consider the imminent era of the Internet of Everything (IoX), where a large number of vehicles and edge smart devices will be interconnected, harnessing their computational capabilities [12]. In this landscape, OTA-FL can play a pivotal role in data-intensive applications, e.g., distributed sensing [13] and autonomous driving [14]. This involves monitoring real-world variables, such as temperature, humidity, and road traffic, making servers more inclined to focus on aggregating different sensor readings to obtain meaningful insights. Furthermore, the utility of OTA-FL can potentially extend to the coordination of unmanned aerial vehicle (UAV) swarms with a central console [15]. OTA-FL can facilitate seamless communication and control between the swarm of drones and the primary console, ensuring efficient and responsive management of the UAVs [16].

The incorporation of OTA can potentially significantly enhance the efficiency of model aggregation in FL. However, the aggregated signal received at the BS inevitably exhibits biases due to channel fading and noise [17]. It is critical to devise suitable transmission and reception strategies to mitigate the impact of channel fading and noise on model aggregation, thereby improving the convergence of OTA-FL systems. Specifically, OTA-FL is confronted with unprecedented challenges, compared to traditional FL. Primarily, OTA-FL conducts analog aggregation within a wireless channel characterized by fading and additive noise [18]. This environment introduces errors during signal reception at the BS [19], setting it apart from the conventional FL process. These errors, in turn, can impact the efficacy of model convergence. Moreover, OTA-FL necessitates achieving signal amplitude alignment among various devices to ensure the accurate recovery of received signals [20]. Consequently, formulating a strategy to guarantee flawless device synchronization represents a distinctive challenge for OTA-FL, particularly in networks characterized by a large number of devices and substantial heterogeneity.

As an emerging technique, the consideration of trust, security, and privacy in OTA-FL has been limited despite their paramount importance for the sustainability and reliability of ML models, as unveiled in this survey. While those aspects have been extensively studied regarding the traditional FL [21], it is not straightforward to apply or extend the existing solutions to OTA-FL due to its distinct communication and aggregation strategy. For example, individual local models are obsolete in OTA-FL. As a consequence, the methods for detecting adversarial local models, e.g., Krum or multi-Krum [22], [23], which require the BS to obtain model updates for each user to compute inter-model Euclidean distances to exclude potential attackers, would no longer be applicable.

Krum and multi-Krum are Byzantine-resilient distributed ML approaches [22]. A score is given to the gradient of every local model, which is the sum of its Euclidean distances to a pre-specified number of its nearest gradients. The pre-specified number depends on the number of adversarial models present. The gradient with the smallest score is used as the global gradient to update the global model [23]. Krum precludes models located farthest from others, which is a typical characteristic of potentially malicious models. As an extension of Krum, multi-Krum precludes multiple most distant local models from the global model [22]. This empowers multi-Krum to effectively identify and isolate multiple potential attackers. However, both Krum and multi-Krum require the BS to know the local model update of each individual user to compute the multi-order moments. They are not applicable to OTA-FL since the BS can only access the aggregated model originating from the participating users.

### 1.2 Contribution of this survey

This survey provides a detailed and comprehensive survey of existing studies on OTA-FL, addresses significant challenges, and outlines potential future research directions. The key contributions of the survey are listed as follows.

• We provide a systematic classification of OTA-FL from a fresh perspective, including single-antenna OTA-FL, multi-antenna OTA-FL, and OTA-FL with the assistance of reconfigurable intelligent surfaces (RISs). We holistically summarize the optimization objectives and algorithms used in the existing works, highlighting their commonalities and pros and cons.
• We delineate the trust, security, and privacy risks confronted by OTA-FL, uncover the gap in the existing literature and point to novel research perspectives crucial for user concerns.
• We identify critical challenges and future directions that need to be addressed, including efficient aggregation, stringent synchronization requirements, abundant data heterogeneity, trustworthiness, and communication-learning metrics.

It is found through this survey that OTA-FL is still in its infancy. Existing studies have typically aimed to minimize the optimality gap of OTA-FL for individual model aggregations under the assumption of ML models with (strongly) convex and smooth loss functions. Little consideration has been given to understanding the impact of channel fading processes on OTA-FL, especially under multi-antenna settings. Moreover, there has been little consideration of the practical implementation of OTA-FL. Challenges, such as model distortion under channel fading, the ineffective OTA aggregation of local models trained on substantially unbalanced data, and the limited accessibility and verifiability of individual local models, have yet to be addressed in the literature.

### 1.3 Comparison with existing surveys

Several recent surveys have delved into the advancements in FL and its related variants. Liu et al. [24] present Vertical FL, a variant of FL characterized by shared samples but distinct client characteristics. This survey effectively reviews algorithms associated with Vertical FL, discussing their progress and challenges in terms of effectiveness, efficiency, and privacy. It also provides valuable insights into the challenges and future directions in this domain. Another noteworthy survey [25] introduces two research focal points within the broader concept of federated computing: federated querying and FL. The concept of federated querying suggests that federated computing should enable secure data owner querying. The survey provides a comprehensive summary of the latest research status in both directions, offering a detailed overview of the ongoing work.

Different from the existing surveys, including [24] and [25], this current paper is dedicated to OTA-FL. The OTA aggregation brings unique challenges, as the aggregated signal often contends with channel fading and noise, necessitating the development of optimization strategies to mitigate aggregation distortion. Handling a large number of devices in the analog domain for waveform aggregation also imposes stringent synchronization requirements, adding complexity to system design. Additionally, the lack of knowledge of the BS about individual user signals renders many traditional security and privacy techniques used in conventional FL ineffective. In this regard, the current paper offers a comprehensive methodological summary and future outlook for addressing these complex issues in OTA-FL.

### 1.4 Organization and notation

**Fig. 2:**
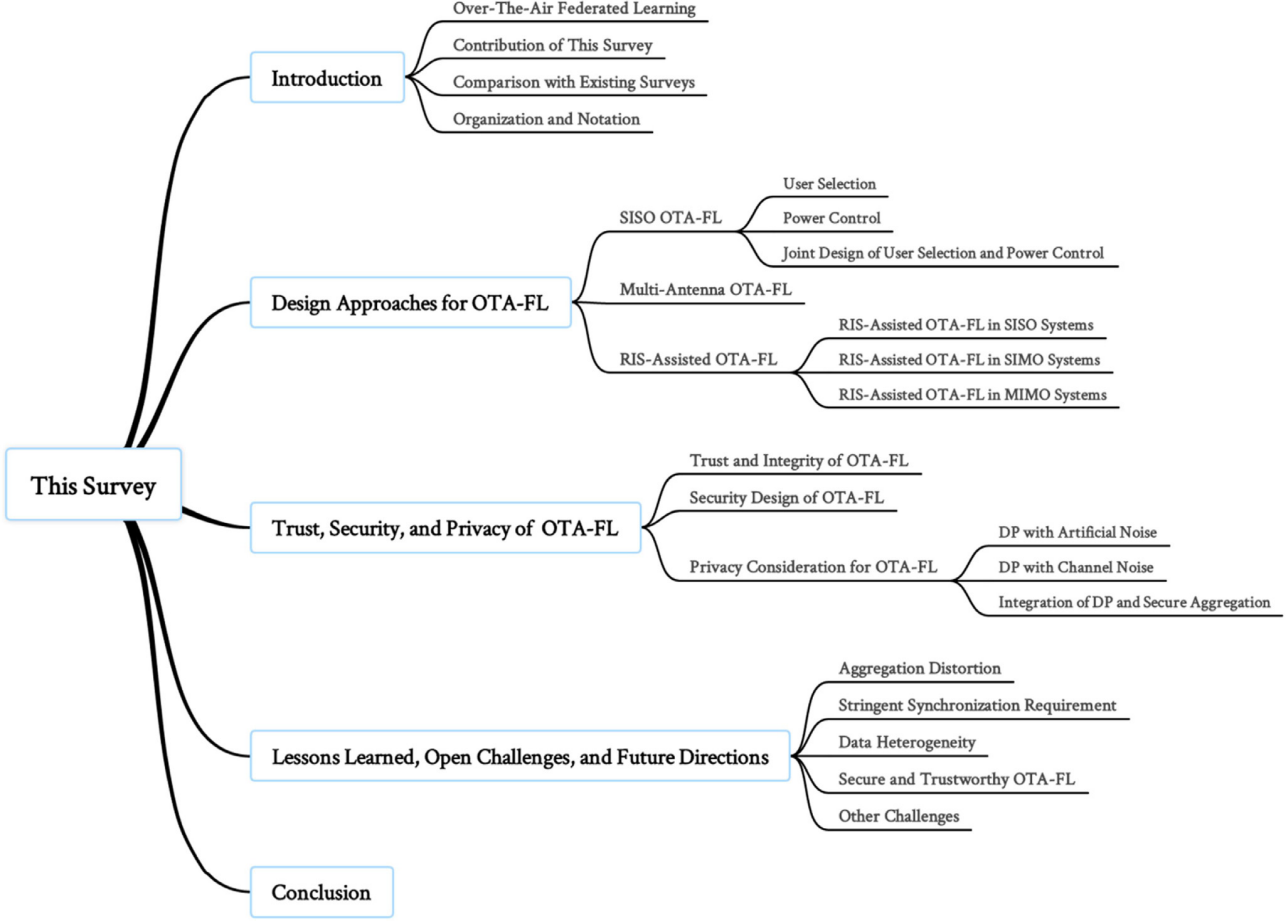
**The taxonomy of the existing studies on OTA-FL**.

The rest of this paper is structured as follows. Section 2 first categorizes and discusses single-input single-output (SISO) OTA-FL system designs from the perspectives of user selection and power control, and then extends the SISO architecture to multiple antenna settings and summarizes strategies addressing optimization problems under multi-antenna settings. The role of RISs in OTA-FL is also discussed, and the corresponding system design strategies are analyzed in the section. Section 3 describes the trust, security and privacy issues and their impacts on OTA-FL. Section 4 points out the challenges and future research directions of OTA-FL, followed by conclusions in Section 5. The abbreviations involved in the paper are collated in Table 1. The taxonomy of the existing studies on OTA-FL is summarized in Fig. 2.

**Table 1:** **List of abbreviations**.

Abbreviation	Full form
6G	The sixth generation
AI	Artificial Intelligence
BEV	Best Effort Voting
BS	Base Station
CSI	Channel State Information
DAB	Decomposed Aggregation Beamformer
DC	Difference-of-convex Function
DNN	Deep Neural Network
DP	Differential Privacy
DRIS	Double-Reconfigurable Intelligent Surface
FL	Federated Learning
GAN	Generative Adversarial Network
IoX	Internet of Everything
I.I.D.	Independent Identically Distributed
ISAC	Integrated Sensing and Communication
LDP	Local Differential Privacy
mmWave	Millimeter Wave
MAC	Multiple Access Channel
MIMO	Multiple-input Multiple-output
ML	Machine Learning
MPC	Multi-Party Computation
MSE	Mean Square Error
MTPDD	Mixed-timescale penalty-dual-decomposition
NIP	Non-linear Integer Programming
NOMA	Non-Orthogonal Multiple Access
NN	Neural Network
Non-I.I.D.	Non-Independent Identically Distributed
OMA	Orthogonal Multiple Access
OMA-FL	Orthogonal multiple access Federated Learning
OTA	Over-the-air
OTA-FL	Over-the-air Federated Learning
PCA	Principal Component Analysis
RIS	Reconfigurable Intelligent Surface
SCA	Successive Convex Approximation
SDR	Semidefinite Relaxation
SGD	Stochastic Gradient Descent
SISO	Single-input Single-output
SNR	Signal-to-noise Ratio
S-CSI	Statistical Channel State Information
UAV	Unmanned Aerial Vehicle
WFL	Wireless Federated Learning
WPT	Wireless Power Transfer

*Notation*: Boldface upper- and lower-cases stand for matrix and vector, respectively; $R^{N}$ denotes the space of all N×1 real-valued column vectors; E[·] takes mathematical expectation; $h^{E}$ denotes the effective channel gain.

## 2 Design approaches for OTA-FL

In this paper, we categorize OTA-FL systems into three categories: SISO OTA-FL, Multi-antenna OTA-FL, and RIS-assisted OTA-FL, according to their different system setups. They each have appropriate application scenarios, pros and cons. In particular, while SISO OTA-FL is relatively easy to configure, it can hardly obtain multiplexing and diversity gains. Multi-antenna OTA-FL can make use of multi-antenna beamforming to improve communication capacity, but the corresponding optimization is often challenging. On the other hand, RIS-assisted OTA-FL utilizes RISs to improve the strength of the received signals, but its standardization and cost control still have a long way to go. Table 2 summarizes the scenarios, pros and cons of three popular OTA-FL setups, namely, SISO OTA-FL, multi-antenna OTA-FL, and RIS-assisted OTA-FL.

**Table 2:** **Comparison between SISO OTA-FL, Multi-Antenna OTA-FL, and RIS-Assisted OTA-FL**.

	SISO OTA-FL	Multi-Antenna OTA-FL	RIS-Assisted OTA-FL
**Senario**	Moderate wireless environments with low channel fading	Complex wireless environments with high communication rate requirements	Wireless environments with large obstacles obstructing the transmitting signal
**Pros**	• Low device requirements • Low optimization complexity	• High spatial diversity gain • High anti-interference capability	• Effective signal strength improvement • Low additional energy consumption
**Cons**	• Low spatial diversity gain • Poor anti-interference capability	• High device requirements • High system complexity	• Difficult to optimize • Configuration and Standardization issues

### 2.1 SISO OTA-FL

We start with an overview of OTA. The primary aim of OTA is to integrate the concurrently transmitted local models or gradients for computing a specific set of nomographic functions [26]:

(1) $$\begin{array}{cc} & \;F\left(d_{1},d_{2},\ldots ,d_{K}\right)=\psi \left(\sum_{k=1}^{K}\varphi_{k}\left(d_{k}\right)\right) \end{array}$$

where $F\left(\cdot \right):R^{K}\to R$ is a nomographic function, $d_{k}\in R$ is a data sample at user k; $\varphi_{k}\left(\cdot \right):R\to R$ and ψ(·):R→R denote the pre-processing function and the post-processing function, respectively. From Eq. 1, we can observe that the nomographic function represents the process of signal transmission and aggregation in OTA-FL, i.e., each user’s local data $d_{k}$ undergoes pre-processing through $\varphi_{k}\left(\cdot \right)$ and is transmitted over wireless channels. The post-processing is subsequently performed through ψ(·) at the BS.

Currently, studies on OTA-FL are still in their infancy, with the majority of studies focused on SISO OTA-FL. Critical issues, such as user selection and power control, have been the primary concerns. There are also studies dedicated to the joint design of user selection and power control to address multiple goods at the same time. In Table 3, we categorize the existing studies on SISO OTA-FL into three classes, including user selection, power control and joint design, and summarize their design goals and optimization strategies, as well as their strengths and weakness.

**Table 3:** **Summary of the existing studies on SISO OTA-FL systems**.

Category	Objective function	Method	Pros	Cons
User Selection	Minimize optimality gap	Lyapunov optimization framework [29]	The framework considers a long-term energy-constrained scenario.	The heuristic objective function makes it difficult to obtain the optimal solution.
		Alternating minimization algorithm and greedy algorithm after problem transformation [30]	Multiple groups of users are within the system, each training one ML task.	Both of the subproblems have suboptimal solutions after problem transformation.
	Maximize the quantity and qualities of devices	Lyapunov optimization framework [31]	The scheme designs a residual feedback mechanism to fully utilize local updates.	Some variables need to be determined in advance, making it difficult to detect their optimal values.
Power Control	Minimize optimality gap	Alternating minimization algorithm after problem transformation [32]	It quantifies the latency advantage of OTA-FL compared to OMA-FL.	The conclusions drawn need to be based on strongly convex cases.
		Lagrange dual method after convexification [33]	It is able to obtain the optimal solutionby convexifying the problem.	The scheme assumes that the user end can only perform one local update.
		Lagrange dual method and subgradient algorithm [19]	The algorithm effectively utilizes S-CSI and can be executed online.	It requires the assumption that fading channels for each user are I.I.D.
	Minimize time-average MSE (derived from optimality gap)	Alternating minimization algorithm [34]	A DNN solver is designed to simplify the process of algorithmic solving.	The interpretability of the DNN solver needs to be further elucidated.
	Minimize error induced gaps (derived from optimality gap)	Feasibility check after problem resolution [35]	It provides performance analysis and collaboration approach for multi-cell FL.	The convergence speed has not been further discussed.
Joint Optimization	Minimize optimality gap	Discrete enumeration algorithm [38]	The algorithm is capable of adapting to multiple local update approaches.	The tightening of the problem solution space leads to suboptimality.
		SDR technique after problem transformation [39]	The algorithm simultaneously considers the impact of uplink and downlink noise.	Suboptimality is introduced when converting the discrete problem into a continuous form.
		Sorting algorithm after problem transformation [40]	The computational complexity is reduced through problem transformation.	The problem-solving process is heuristic in nature.

#### 2.1.1 User selection

In SISO OTA-FL systems, several factors, such as the size of local data and channel quality, often influence the significance of local updates for each user. In scenarios where aggregating data from all users is not feasible, selecting the most “important” users for training participation becomes necessary, as this selection can significantly impact the system’s performance. However, the user selection problem is typically a discrete non-convex problem, which necessitates the search for efficient solving methods [27].

A trade-off is proposed by Zhu et al. [28], where the authors suggest that aggregating training data from a more significant number of devices per round can expedite convergence. However, this approach may also lead to increased aggregation error due to the inclusion of devices with poorer channels. The work presented by Sun et al. [29] addresses a user scheduling problem considering the cumulative energy budget of each user over T rounds. By analyzing the convergence performance, the authors design an estimated drift-plus-penalty algorithm using Lyapunov optimization. An estimation method is employed to forecast the norm of local gradients to overcome the challenge of unknown communication energy.

In the context of multiple parallel OTA-FL in cellular networks, discussed in [30], the authors focus on a scenario where a server handles multiple model training tasks from distinct groups utilizing identical radio resources. They define an optimization problem to simultaneously optimize receiver combiner vectors and user selection to minimize the time-varying convergence upper bound. To tackle this problem, they decompose it into two sub-problems, which are addressed using a successive convex approximation (SCA) method and a greedy algorithm. Du et al. [31] propose a dynamic device scheduling framework using channel inversion-based power control. They design a measurement factor inspired by the convergence upper bound, which takes into account both the quality and quantity of selected users as the optimization objective. The problem is then solved using Lyapunov optimization.

#### 2.1.2 Power control

The design of power control strategies plays a crucial role in mitigating the impact of fading and ensuring robust received signal strength for users in weak channels at the BS, thereby enhancing the performance of OTA-FL systems. Existing research primarily focuses on power control issues through the minimization of the optimality gap and obtaining the optimal power distribution factor. Most existing works, such as [32], [33], and [34], aim to achieve user power control by minimizing the optimality gap in each round.

In [32], an optimality gap minimization method is proposed to address aggregation errors characterized by mean square error (MSE). This is accomplished by optimizing transmission power and a denoising factor, and the problem is solved using an alternating minimization algorithm. Similarly, Cao et al. [33] obtained a power control strategy by minimizing the optimality gap while considering unbiased aggregation constraints. The formulation incorporates both average and maximum power constraints for each user, and convex reformulations are employed with structured optimal solutions. Zou et al. [34] derive the effects of model aggregation errors accumulated among communication rounds using the upper limit of the time-average norm of model parameter gradients. Power control and transceiver policies are obtained by minimizing the derived upper bound through an alternating optimization algorithm. Wang et al. [35] investigated OTA-FL in a multi-cell wireless network where different training tasks are performed in each cell. A convergence analysis is carried out by taking inter-cell interference, and a problem is formulated to minimize the error gap across all cells while adhering to power constraints.

In order to decrease the overhead of channel estimations in OTA-FL, Jing et al. [36] put forward two OTA-FL schemes that utilize statistical channel state information (CSI), known as S-CSI. These schemes aim to reduce the associated cost of channel estimations [37]. The convergence bound of OTA-FL has been analyzed by Sery et al. [18], with a specific focus on the impact of channel fading on the distortion of OTA-FL. A few interesting insights are unveiled:

• The per-round convergence upper bound depend primarily on the mean and variance of channel fading states, $\mu_{h}$ and $\sigma_{h}^{2}$.
• With the increase of $\mu_{h}$ and the decrease of $\sigma_{h}^{2}$, this convergence upper bound can effectively shrink when the ML model has strongly convex or convex loss functions. Meanwhile, a larger $\mu_{h}$ and a smaller $\sigma_{h}^{2}$ can counteract the side effects caused by channel noise.

In light of the insights, an optimal adaptive power control scheme has been recently proposed to combat the channel-induced model distortion of OTA-FL by leveraging S-CSI by Yu et al. [19]. The objective of this scheme is to minimize the optimal gap of OTA-FL under any unknown channel fading conditions, as given by

(2) $$\begin{array}{cc} & \;\min_{\left\{\rho (h)\geq 0,\forall h\right\}}G\left(\mu_{h^{E}},\sigma_{h^{E}}^{2}\right)\text{s.t.}\;E_{h}\left[\rho^{2}\left(h\right)\right]\leq P_{0} \end{array}$$

where G(·) is the optimality gap of the convergence, and ρ(h) specifies the power control policy. Based on the findings of Sery et al. [18], this optimization problem can be translated to essentially construct an efficient channel with a large mean $\mu_{h^{E}}$ and a small variance $\sigma_{h^{E}}^{2}$. By transforming the problem into a nested optimization problem and solving it with the Lagrange dual method, the optimal power control policy can be found to exhibit the following structure:

(3) $$\begin{array}{cc} & \;\rho^{*}\left(h\right)=\frac{h(2\mu_{h^{E}}^{*}-\nu^{*})}{2(h^{2}+\lambda^{*})},\;\forall h \end{array}$$

where the optimal dual variables $\lambda^{*}$ and $\nu^{*}$ is obtained using a subgradient ascent method, and $\mu_{h^{E}}^{*}$ is obtained efficiently using a one-dimensional search method.

Following this policy, at each communication round, a user only needs to know its current channel state and update the dual variables accordingly. Even without the knowledge of future channel variations, the policy has been proven to provide a long-term optimal power control strategy under independent and identically (I.I.D.) channel environments. The policy can also be extended in the situation where even the channel statistics, i.e., $\mu_{h^{E}}$ and $\sigma_{h^{E}}^{2}$, are unknown *a-priori*. The policy can estimate the channel statistics on-the-fly based on the accumulated historical observations of the channels.

#### 2.1.3 Joint design of user selection and power control

There has been a growing interest in simultaneously considering and optimizing user selection and power control for OTA-FL, recognizing that joint design may not always guarantee optimality for each objective due to transformations or decompositions of the original problems. However, this joint approach can enhance system robustness by improving the system design from multiple perspectives.

For Fan et al. [38] and Guo et al. [39], an optimal co-design strategy for user selection and power control is obtained by minimizing the optimality gap, leveraging system convergence analysis. This joint strategy takes into account both user selection and power control to achieve improved performance. Furthermore, Chen et al. [40], extend the consideration to include energy harvesting strategies on the user side. They formulate a non-convex non-linear integer programming (NIP) problem to optimize client selection on a per-training-round basis, addressing the energy harvesting aspect of OTA-FL in addition to user selection and power control optimization.

### 2.2 Multi-antenna OTA-FL

Recently, there has been a growing interest in exploring more complex scenarios involving multiple antennas at the transmitters and/or receivers in OTA-FL. In such cases, joint optimization of transmitter and receiver beamforming, along with user selection and power control, becomes crucial to achieve enhanced performance. Compared to the SISO OTA-FL systems, the design of OTA-FL systems with multiple antennas is still at an early stage, offering significant opportunities for further development and exploration.

**Fig. 3:**
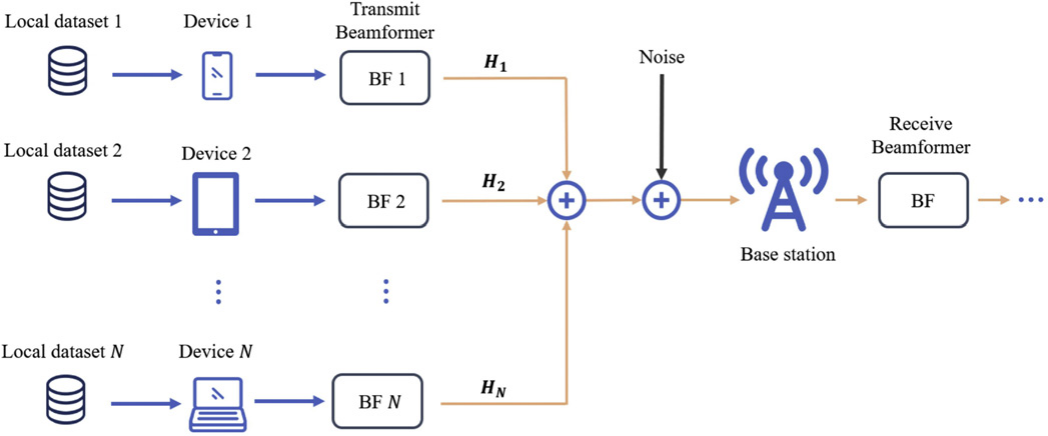
**An illustration of multi-antenna OTA-FL system, where**$H_{n}$**denotes the channel matrix of Device**n**.** To fully utilize the benefits of multiple antennas, a joint design approach is commonly adopted to optimize user selection, power control and beamforming vectors.

Given the strong interdependence among variables in multi-antenna OTA-FL systems, existing studies predominantly adopt a joint design approach, aiming to optimize variables such as user selection, power control, and beamforming vectors, which are highly coupled, as illustrated in Fig. 3. These joint design approaches are motivated by the need to exploit the benefits of multiple antennas in OTA-FL systems, such as improved spectral efficiency and enhanced communication reliability. By jointly optimizing user selection, power control, and beamforming, the performance of multi-antenna OTA-FL systems can be significantly improved, thereby unlocking the potential of utilizing multiple antennas effectively for distributed learning tasks. In Table 4, we summarize the efforts made by existing studies in exploring MIMO OTA-FL systems and summarize their respective optimization objectives and methods, as well as their strengths and limitations.

**Table 4:** **Summary of the existing studies on Multi-Antenna OTA-FL systems**.

Objective function	Method	Pros	Cons
Minimize the MSE of received signal	SCA-based optimization algorithm [41]	It can achieve performance close to that of the fully-digital beamforming.	SCA approach is employed, which leads to suboptimal solutions.
	Equivalence between original problem and nested sub-problems [42]	The algorithm can lead to a global optimal solution.	The computational complexity increases sharply with the number of antennas.
	Grassmann manifold approximation [43]	The algorithm effectively reduces computational workload compared to SDR approach.	Due to the limitations on nomographic functions, OTA channel feedback is not applicable to traditional multi-user MIMO cases.
	SDR based algorithm [44]	The system is capable of simultaneously fulfilling communication and sensing.	The embedding of sensing results in a partial degradation of communication performance.
	One-dimensional search algorithm [45]	It achieves low-latency channel feedback based on OTA characteristics.	The original optimization problem needs several approximation, which causes suboptimality.
	Approximate equivalence of solving subproblems [46]	The scheme is designed to operate without explicit S-CSI requirements.	The heuristic algorithm can only obtain a local optimal solution.
Minimize MSE and maximize quantity of devices	DC programming after problem transformation [47]	Effective user selection is achieved by obtaining the low-rank sparse structure of the problem.	The SDR-based solution results in high computation complexity.
	Greedy algorithm based on matching pursuit [48]	The computational complexity is effectively reduced.	The loss function needs to be strongly convex which leads to certain limitations.
Minimize optimality gap	Alternating optimization and fractional programming [49]	The scheme enables to alleviate system straggler issues.	The strong convexity assumption for FL loss functions has certain limitations.
	Feasibility test with bisection search [50]	The pratical computation overhead of the algorithm is low.	The algorithm requires mild conditions and slack transformations.

In multi-antenna OTA-FL systems, the prevailing approach for optimization is centered around minimizing MSE. For instance, Zhai et al. [41], the authors tackle the problem of optimizing users’ transmit beamforming and the BS’s receive beamforming to minimize the MSE of the received signals. To address the challenges posed by highly coupled variables, they propose an alternating minimization algorithm that jointly optimizes the transmit and receive beamforming vectors. However, recent studies suggest that exploring alternative communication-learning representation objectives may offer a more promising approach than solely focusing on MSE. While MSE minimization remains the mainstream practice, researchers are starting to recognize the potential benefits of adopting different optimization objectives.

In the context of MIMO OTA-FL systems Li et al. [42] considers the optimization of wireless power transfer (WPT). They reformulate the non-convex MSE minimization problem into two nested sub-problems, making it tractable and enabling efficient solutions. Another approach is presented by Zhu et al. [43], where equalization and channel feedback techniques are designed to improve OTA computation capabilities. The objective is to minimize computation MSE, and the authors propose an equalization strategy based on differential geometry and a channel feedback mechanism to facilitate OTA computation functions. Furthermore, there are studies that explore the integration of OTA with other systems. For Li et al. [44], OTA is combined with an integrated sensing and communication (ISAC) system to leverage the advantages of both sensing and OTA computation. The optimization objective in this case is to minimize radar sensing MSE while maintaining sensing accuracy.

In the context of IoT networks, Wen et al. [45] propose a MIMO OTA scheme for networks with clustered multi-antenna sensors and a receive array at the base station. They introduce an optimal receive beamformer, known as the decomposed aggregation beamformer (DAB), which utilizes a decomposed architecture to reduce the channel dimension and perform joint equalization. Additionally, they propose a low-latency channel feedback framework that leverages OTA to enable simultaneous channel feedback from the sensors.

To address signaling overhead and power consumption concerns, Chen et al. [46] present a joint optimization scheme that minimizes MSE through the optimization of transmit beamformers and hybrid combiners. This scheme achieves local stable convergence by dividing time slots and optimizing two subproblems: short-term and long-term optimizations. For Yang et al. [47], a joint approach incorporating user selection and receiving beamforming design is utilized to achieve fast model aggregation. This approach employs a difference-of-convex-functions (DC) representation to improve sparsity and accurately detect the fixed-rank constraint. Similarly, an efficient user selection scheme is achieved by Bereyhi et al. [48] by maximizing the number of selected devices and minimizing the aggregation error. A greedy method based on matching pursuit is designed to reduce computational complexity and preserve training performance of the DC method achieved by Yang et al. [47].

While the majority of existing studies in multi-antenna OTA-FL focus on MSE minimization, there are only a few studies that explore minimizing the optimality gap. For example, confronting multiple concurrent FL tasks, Zhong et al. [49] propose a strategy to jointly optimize transceiver beamforming and user selection by minimizing an upper bound under a MIMO OTA multi-task FL framework. They develop an algorithm based on an alternating optimization framework with low complexity, which addresses the optimization problem while alleviating the straggler problem by aligning the uploaded gradients at the BS. Ma et al. [50] investigate model sparsification and stochastic compression to mitigate inter-task interference in OTA multi-task FL. Turbo compressed sensing is utilized at the receiving end to reconstruct the model aggregations of different tasks. By minimizing the optimality gap, a distinct inter-task power control problem is studied to further improve overall system performance. Such works have made an effective attempt to deploy OTA-FL in complex realistic scenarios.

### 2.3 RIS-assisted OTA-FL

**Fig. 4:**
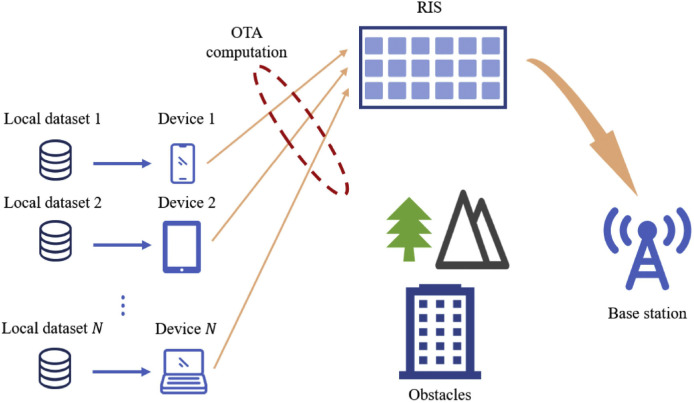
**An illustration of RIS-assisted OTA-FL system, where the RIS alters the propagation direction of reflected signals by manipulating the phase of incident signals, thereby enhancing the received signal strength at the BS**.

In the context of the envisioned development of 6G, the integration of RIS technology is gaining significant attention and is considered a promising solution for OTA-FL systems. RIS, as a cost-effective passive device, consists of multiple reflective elements capable of adjusting the phase shift of incoming signals, consequently altering the propagation direction of the reflected signals [51], [52], [53], [54], as illustrated in Fig. 4. This unique capability of RIS enables the superimposition of the direct link signal, effectively mitigating signal energy attenuation resulting from obstacles such as buildings [55].

RIS holds great potential for improving the performance of OTA-FL systems by overcoming signal propagation challenges. By strategically deploying RIS in the wireless environment, it becomes possible to enhance signal strength and quality, leading to improved communication efficiency and higher reliability [56], [57], [58]. The use of RIS technology can effectively address issues related to signal attenuation and multipath fading, which are common obstacles in wireless communications. Additionally, RIS can offer flexibility in optimizing signal propagation paths, thus enabling better coverage and reduced interference in OTA-FL systems.

Several studies have focused on incorporating RIS into OTA-FL frameworks, as delineated in the following. We provide a detailed summary of OTA-FL systems assisted by RISs in Table 5, where it is categorized into the settings of SISO, SIMO, and MIMO, according to the number of antennas at the transmitters and the receiver. The optimization objectives, variables, and methods of the systems, as well as their strengths and limitations, are presented accordingly.

**Table 5:** **Comparative summary of the existing studies on RIS-assisted OTA-FL systems**.

Antenna Setting	Joint Design	Objective function	Method	Pros	Cons
SISO	Transceiver design and RIS phase shifts	Minimize the MSE of received signal	Alternating minimization algorithm [59]	The subproblems have closed-form solutions after problem decomposition.	Specific trade-offs regarding the number of RISs have not been provided.
SIMO	User selection, RIS phase shifts and beamformer at BS	Maximize the number of scheduled users	Alternating minimization algorithm [60]	The device selection process achieves performance close to that of an exhaustive approach.	The algorithm achieves better performance but at the cost of higher computational complexity.
		Minimize optimality gap	SCA-based optimization algorithm [61]	The proposed algorithm has low computational complexity.	The assumption is made that the loss function is strongly convex.
	Tranmit scalar, RIS phase shifts, beamforming vectors and denoising factor at BS	Minimize the MSE of received signal	Alternating minimization algorithm [62]	The algorithmis able to reduce computational time while achieving similar performance.	The approximation of the problem leads to suboptimal solutions.
	Transmit power, RIS passive beamforming and beamformer at BS		Penalty-dual-decomposition algorithm [63]	The system performance is able to match the theoretical upper bound.	The case of large-scale fading has not been mentioned.
	RIS phase shifts and beamformer at BS		Alternating minimization algorithm [64]	The scheme achieves more efficient multi-cell interference management.	The computational complexity of the algorithm has not been mentioned.
	Transmit scalar, RIS passive beamforming and beamformer at BS		Alternating minimization algorithm [65]	Theoretical proofs demonstrate the performance advantages of DRIS over a single RIS.	The proposed algorithm exhibits weaker performance compared to SDR-based approaches.
	Beamforming vectors,denoising factor,power control and passive beamforming		Alternating minimization algorithm [66]	Closed-form solution structures can be obtained for part of the problems.	No theoretical justification is provided for the superiority of DRIS over a single RIS.
	User selection, RIS phase shifts, decoding vector, and power control	Minimize power-delay product	Problem decomposition [67]	The algorithm is able to utilize RISs to reduce energy consumption.	The algorithm cannot guarantee optimality through iterative solving of individual subproblems.
MIMO	Transmission distortion, beamforming vectors, and RIS phase shifts	Minimize the MSE of received signal	Alternating minimization algorithm [68]	The algorithm does not require the assumption of strongly convex loss functions.	The computational complexity of the algorithm has not been mentioned.

#### 2.3.1 RIS-assisted OTA-FL in SISO systems

In OTA-FL systems incorporating RISs, comprehensive optimization of multiple variables is necessary, including RIS configurations, user selection, and power control. However, these optimization problems can be challenging due to their non-convex nature and the close coupling of variables. To address performance bottlenecks under poor propagation channel conditions, OTA-FL systems with RISs are designed by Zhang et al. [59] with a single antenna deployed on both the BS and device side. It focuses on the co-design of transceivers and RIS phase shifts to minimize aggregation errors. However, the practical performance limitations arising from the single antenna assumption at the BS have spurred the investigation of RIS-assisted OTA-FL systems employing multiple antennas.

#### 2.3.2 RIS-assisted OTA-FL in SIMO systems

The predominant focus in current works lies in the deployment of multiple antennas at the BS in RIS-assisted OTA-FL systems. Introducing multiple antennas provides increased degrees of freedom but also leads to a larger number of variables that need to be optimized.

Wang et al. [60] propose the use of RISs to mitigate the impact of fading channels and enable reliable model aggregation. It is important to note that increasing the number of reflecting elements in the RIS can aggregate more devices for training and improve performance. However, this also leads to increased channel estimation overhead. Thus, a co-design of power control and resource distribution is required to strike a balance. Similarly, Liu et al. [61], the trade-off is investigated between communication and learning, highlighting the essentiality of RIS deployment. The authors propose an algorithm that jointly optimizes user scheduling, receiver beamforming vectors, and RIS phase shifts to achieve the desired performance.

Similar to Zhang et al. [59] and Fang et al. [62] aims to address performance bottlenecks under poor propagation channel conditions by designing transmit power of devices, transceivers, and RIS phase-shifts. For Zhai et al. [63], a mixed-timescale penalty-dual-decomposition (MTPDD) algorithm is proposed to reduce signaling overhead caused by a large number of RIS elements. The algorithm jointly minimizes the MSE of computation over time while mitigating signaling overhead. The potential of OTA-FL in multi-cell networks is explored by Li et al. [64] through the utilization of RISs. The authors propose an alternating minimization scheme to optimize receiving beamforming vectors and RIS phase shifts jointly. Furthermore, for Zhai et al. [65], the concept of a double-RIS (DRIS) OTA-FL system is introduced, where a pair of RISs are placed at the users’ and BS ends, respectively. This configuration addresses the problem of unavailable direct links due to obstacles. It is demonstrated that the MSE performance of the DRIS system surpasses that of single RIS systems when a substantial number of RIS elements and receiving antennas are deployed. Similarly, for Li et al. [66], a DRIS-assisted OTA-FL system with multiple antennas is proposed to enhance the channel quality. The objective is to minimize MSE by jointly optimizing receiving beamforming vectors, denoising factor, power control, and passive beamforming design. By considering all these variables together, the system performance can be improved.

Hu et al. [67] formulate an energy minimization problem that aims to jointly optimize user selection, phase shifts, decoding vectors, and power control. To handle the complexity of the problem, it is decomposed into several subproblems and solved iteratively.

While it may be challenging to obtain the global optimum in the works mentioned above, significant performance gains have been achieved through the rational use of RISs and the design of optimization algorithms. These studies contribute to the exploration and advancement of OTA-FL systems, leveraging the benefits of RIS technology.

#### 2.3.3 RIS-assisted OTA-FL in MIMO systems

Some works also consider RIS-assisted OTA-FL in MIMO systems. In [68], both the BS and user terminals are equipped with multiple antennas to facilitate concurrent model transmission over a millimeter wave (mmWave) network. The objective is to minimize transmission distortion. Thus, joint optimization of RIS phase shifts and beamforming vectors is considered to achieve this goal.

A critical challenge in RIS-assisted OTA-FL systems is the assumption of perfect instantaneous CSI in existing studies. However, obtaining accurate CSI can be computationally expensive or even infeasible, particularly in scenarios where the system is mobile and the channel exhibits high dynamics. This limitation poses a significant hurdle in optimizing the performance of such systems. The integration of RIS technology in OTA-FL systems represents a promising avenue for research and development. It has the potential to revolutionize wireless communications by mitigating signal attenuation and improving overall system performance. As the exploration of RIS in OTA-FL continues to expand, further advancements and optimizations are expected to unlock its full potential in realizing efficient and reliable next-generation wireless networks.

## 3 Trust, security, and privacy of OTA-FL

FL has made significant strides in improving privacy compared to centralized learning, as it enables local processing of raw data. However, the baseline OTA-FL model still lacks a formal guarantee of trust, security, and privacy [69]. While these terms are often used interchangeably in existing literature, it is important to highlight their distinct meanings.

In the context of OTA-FL, trust, security, and privacy take on heightened significance, significantly impacting its operation and applied applications. Trust assures all stakeholders that an FL process unfolds as expected [70]. This is crucial when multiple users contribute their local model updates over the air. Trust also ensures that participants have confidence in the accuracy and fairness of an aggregation process, leading to collaborative and productive FL scenarios. Security is a paramount concern in OTA-FL. With model updates transmitted over wireless channels, it is imperative to protect these updates from unauthorized access, tampering, or interception [71]. Security measures, such as encryption and secure communication protocols, safeguard the integrity and confidentiality of the model updates, defending against potential threats and ensuring the reliability of OTA-FL [72]. Privacy is another dimension of OTA-FL. Not only does it involve protecting the models, but also the privacy of the individual user data used to train those models. OTA-FL must ensure that users’ sensitive data remains private during model aggregations [73]. Techniques, such as differential privacy (DP) [74], may help balance the trade-off between the privacy and utility of OTA-FL. Trust, security, and privacy are not only foundational but also essential for the successful application of OTA-FL. They ensure that participants can collaborate with confidence, protect sensitive information, and maintain the privacy of user data, advancing the efficiency and effectiveness of OTA-FL.

To ensure trust, security, and privacy in OTA-FL, various mechanisms have been proposed and investigated. Researchers have explored different approaches to develop OTA-FL systems that preserve these crucial aspects. These mechanisms aim to establish a robust framework that instills confidence in the system’s operations, safeguards against unauthorized access and malicious attacks, and protects the privacy of sensitive information. By emphasizing the distinctions between trust, security, and privacy, researchers can better understand the multifaceted nature of OTA-FL systems and design comprehensive solutions. By integrating these mechanisms into the OTA-FL model, it becomes possible to establish a trustworthy, secure, and privacy-preserving framework that meets the requirements of various stakeholders involved in the collaborative learning process. Table 6 presents a summary of the existing studies on the trust, security, and privacy of OTA-FL, where the design objectives and security or privacy guarantees are systematically reviewed.

**Table 6:** **Summary of the existing consideration of Trust, Security and Privacy in OTA-FL systems**.

Category	Counteracting	Guarantee	Method	Description
**Trust**	This area is important but current research is limited.			
**Security**	Data poisoning and model poisoning (e.g., backdoor attacks)	Byzantine-resilience	Geometric median aggregation	Introduce Weiszfeld’s algorithm to lower computation complexity. [78]
				Group devices and assign transmit slots before aggregation. [87]
			Best effort voting	Allow devices to transmit with maximum power to defend Byzantine attacks. [88]
**Privacy**	Membership inference, property inference and model inversion	Differential privacy	Using inherent channel noise	Minimize the optimality gap with privacy obtained “for free” from channel. [90]
				Develop a power control strategy to preserve DP from channel noise. [91]
			Adding artificial noise	Combine the local gradients linearly with artificial Gaussian noise. [89]
				Propose misaligned power allocation to enhance the system SNR. [92]
				Add spatially correlated perturbation noise to the local updates. [93]
				Distribute the noise generating process to defend pilot attacks. [94]
				Propose a privacy-preserving variant of the second-order method ADMM. [95]
				Reduce the update dimension and enhance the communication efficiency. [96]
		Differential privacy secure aggregation		Add pairwise cancellable noise to thwart external eavesdroppers. [97]
				Assign part of devices to send noise to degrade the SNR of eavesdroppers. [98]

### 3.1 Trust and integrity of OTA-FL

Trust is a concept that reflects the level of control and confidence that an entity has in another entity. It can also be seen as an outcome resulting from advancements in achieving security and privacy objectives [75], [76]. In the context of FL, trust plays a significant role. For example, enterprises must trust network operators when granting consent for the collection of their data in FL. However, the abundance of valuable private data introduces concerns regarding algorithms that aim to predict specific business states.

The lack of trust among participants in FL remains an ongoing challenge. In an effort to address this issue, FLChain is proposed by Bao et al. [77]. FLChain is a decentralized, publicly auditable, and healthy FL ecosystem that incorporates trust and incentives. It aims to create an environment where participants can trust the system and each other. However, to date, no research specifically focuses on establishing trust in the field of trustworthy OTA-FL.

In order to foster trust in OTA-FL systems, it is crucial to explore mechanisms that enhance transparency, accountability, and verifiability. These mechanisms can include decentralized architectures, public auditing, and incentives for participants. By incorporating these elements into the design and implementation of OTA-FL frameworks, researchers can work towards establishing trust among the involved entities and ensuring the reliability and security of the collaborative learning process.

### 3.2 Security design of OTA-FL

The security concerns associated with OTA-FL primarily revolve around the potential for poisoning attacks, where malicious participants aim to compromise the federated learning process. These attacks can be classified into two main categories:

• *Data Poisoning*: One example of a data poisoning attack is the label-flipping attack, as described by Fung et al. [79]. In this type of attack, the adversary maintains the same features in their training sample but flips the corresponding label to a different class. The adversary aims to corrupt the training process and influence the learned model by introducing such maliciously labeled data [80].
• *Model Poisoning*: Adversaries in OTA-FL have the ability to manipulate local model updates before transmitting them to the server. This manipulation can lead to various outcomes, such as causing misclassifications or implanting hidden backdoors [81] within the global model. It has been observed that in FL scenarios, model poisoning attacks tend to be more effective compared to data poisoning attacks [82].

These poisoning attacks pose significant security risks to OTA-FL, as they can undermine the integrity and reliability of the federated learning process. Mitigating these threats requires robust security measures and techniques to detect and prevent malicious behavior from compromising the OTA-FL system.

Indeed, distance-based defense mechanisms are one of the common defense mechanisms in FL. It detects and discards malicious updates by comparing the distance (e.g., Euclidean distance) between users’ local model updates [83]. Blanchard et al. [22] propose Krum and its extension Multi-Krum. Krum is able to exclude models that are furthest away from the other models, which is a typical characteristic of malicious models. Multi-Krum excludes multiple models that are furthest away from other models, which allows for the detection, identification, and exclusion of multiple potential attackers.

FoolsGold is designed by Fung et al. [84], which utilizes the cosine similarity between models as as a discerning metric to identify malicious updates. In FoolsGold, anomalous updates typically exhibit a higher cosine similarity among themselves, leading to their assignment of lower aggregation weights. For Cao et al. [85], a system called Sniper is introduced, which involves the construction of a graph by the BS. In this graph, the vertices represent local models collected from various users. To determine if an edge should exist between these vertices, Euclidean distance is employed as a measure. Once the edges are established, a corresponding set of local models is chosen for aggregation. For Wan et al. [86], MAB-RFL follows a strategy akin to Sniper. Initially, it identifies updates that share similar directions, effectively discarding those susceptible to collusive attacks. Subsequently, Principal Component Analysis (PCA) is employed to extract crucial parameters from these updates. This transformation into a lower-dimensional parameter space enhances the ability to distinguish between benign and malicious updates.

While the distance-based defense mechanisms mentioned above prove effective in traditional FL, the inherent limitation of OTA-FL, i.e., the lack of access to individual users’ local model updates, poses a significant challenge for the direct application of these methods. Consequently, there is a pressing need for more extensive research to develop security defense mechanisms tailored specifically to the unique requirements and constraints of OTA-FL.

**Fig. 5:**
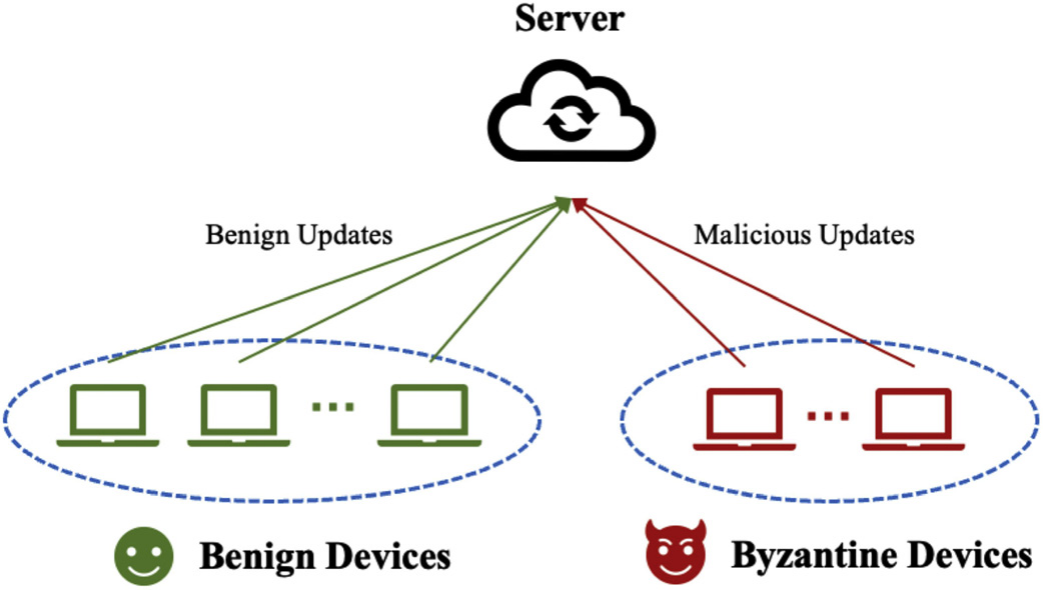
**An illustration of the OTA-FL framework with a server, benign devices, and Byzantine devices, where the Byzantine devices launch attacks to contaminate the benign devices through the global model aggregation at the server**[78].

The simplicity of OTA-FL procedures can render the learning process vulnerable to intentional poisoning attacks by adversaries. In recent years, there has been growing interest in Byzantine attacks, where malicious devices aim to disrupt FL convergence or steer it towards a poisoned model, as illustrated in Fig. 5. Therefore, it is crucial to design secure OTA-FL systems that can effectively counteract these attacks. Currently, only a few works specifically address Byzantine attacks in the context of OTA-FL, which are described below.

For instance, Sifaou et al. [87] propose ROTAF, a novel transmission and aggregation approach that enhances the robustness of OTA-FL against Byzantine attacks in the case of I.I.D. data distribution. In ROTAF, participating devices are divided into different groups during each global training round, and each group is assigned a separate transmit time slot. The local updates from different groups are aggregated using geometric median aggregation. When dealing with non-I.I.D. data, a resampling step is performed before applying geometric median aggregation. The authors provide theoretical convergence analysis of ROTAF under both I.I.D. and non-I.I.D. data assumptions, demonstrating its ability to converge to within a range of the optimum at a linear rate when the number of groups exceeds twice the number of attacks. Numerical results show that ROTAF exhibits robustness against various forms of Byzantine attacks compared to the basic averaging OTA-FL approach.

Huang et al. [78] focus on reducing the computation complexity of Byzantine-resilient OTA-FL. They address the challenge posed by the complexity of solving a convex problem in the commonly-used geometric median aggregation approach when the model parameter dimension is large. To overcome this, they adopt the improved Weiszfeld algorithm to calculate the smoothed geometric median. By leveraging the additive structure of the Weiszfeld algorithm, which can be combined with OTA, they propose a secure aggregation approach that is jointly designed for computation and communication in OTA-FL. Fan et al. [88], highlight the limitations of the widely-used channel inversion method in OTA-FL for defending against Byzantine attacks. They introduce a novel policy called best effort voting (BEV), which integrates local stochastic gradient descent (SGD) to ensure safe aggregation. BEV allows users to transmit their local gradients with maximum power, maximizing the deterrent effect on OTA-FL convergence. The authors analyze the convergence of BEV and demonstrate its superiority over popular channel inversion methods, particularly under strong adversarial environments.

These works represent important contributions to addressing Byzantine attacks in OTA-FL, providing techniques and mechanisms to enhance the security and resilience of the learning process against malicious behavior.

### 3.3 Privacy consideration for OTA-FL

While FL provides a distributed approach to model training without the need to share local data with the BS or other participants, it is important to note that FL is not immune to privacy concerns [99]. Recent research has demonstrated that FL is susceptible to inference attacks [100], which can potentially lead to the recovery of local training data. This vulnerability arises because the model or gradient updates obtained from local data can unintentionally reveal additional information about the underlying features in the data that were not intended to be disclosed.

In the context of OTA-FL, attacks that exploit this privacy vulnerability can be categorized as follows:

• *Membership Inference*[101]: Adversary could test whether a specific data partition of a device has been utilized for training a model. On the FourSquare location dataset, the authors show that a malicious server can 99% convincingly tell if particular location metadata is used to train a classifier.
• *Property Inference*[102]: Adversary could test if a specific partition of data with certain properties is contained in the data of a device. It is significant to emphasize that this property may not be directly associated with the primary objective.
• *Model Inversion*[103]: Adversary could reconstruct an input sample of the training data of a device based on the local updates. One sample of the training dataset is generated, which should be private with a generative adversarial network (GAN).

Privacy protection measures, such as homomorphic encryption and secure multi-party computation, have found applications in the realm of FL. Homomorphic encryption, for instance, offers the ability to aggregate ciphertexts without requiring prior decryption [104]. In this process, during training, each device encrypts its model update using a public key and transmits the ciphertexts to the BS for aggregation. Subsequently, the BS redistributes the result to devices for decryption using their private keys. This enables the devices to update their local models and proceed to the next iteration. As only encrypted local model updates are uploaded, the BS gains no insight into the transmitted data, ensuring privacy.

To mitigate the substantial communication and computational overhead associated with homomorphic encryption in FL, BatchCrypt is developed by Zhang et al. [105]. BatchCrypt encodes batches of quantized gradients into long integers and encrypts them as a whole. New quantized coding schemes and gradient trimming techniques are devised to further reduce overhead. Additionally, Zhang et al. [106] propose Dubhe, which leverages homomorphic encryption to enable active user participation in training while preserving privacy. However, in the context of OTA-FL, which necessitates waveform superposition in the analog domain, the integration of digital cryptographic operations required by homomorphic encryption becomes a formidable challenge.

On the other hand, secure multi-party computation serves as a protocol that facilitates collaborative computation among multiple participants without disclosing the original data, ensuring the security of the results. Founded on a chained secure multi-party computation scheme, Chain-PPFL is developed in [107] for traditional FL systems. Chain-PPFL employs two mechanisms, single-masking and chained communication, to cater to the need for safeguarding information exchange and information chaining processes, respectively. Kanagavelu et al. [108] propose a multi-party computation-enabled FL solution for traditional FL systems. To tackle the high communication costs, a two-phase scheme is put forth, where model aggregation services supporting the Multi-Party Computation (MPC) are extended to additional participants via a small elected committee. Unfortunately, the substantial communication overhead and modulation format challenges associated with MPC still hinder its direct application in OTA-FL.

**Fig. 6:**
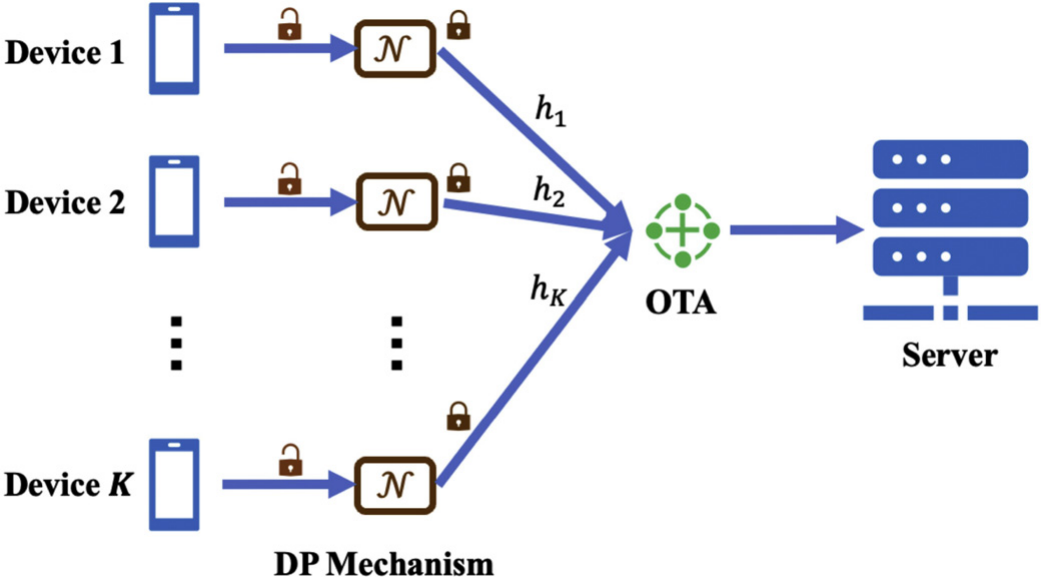
**An illustration of the OTA-FL framework with DP, where the communication between devices and the server must adhere to DP requirements for each user and be obfuscated with artificial Laplacian or Gaussian noise**[89].

It is crucial to design private-by-design OTA-FL systems that are inherently against inference attacks. To this end, differential privacy (DP), one of the perturbation-based methods, has been adopted as a standard solution for preserving privacy in OTA-FL, as illustrated in Fig. 6. DP serves as a robust standard for ensuring privacy in distributed systems [109]. A randomized mechanism N:D→K, where D denotes domain and K denotes range, is of (ϵ,δ)-DP, on condition that for arbitrary two adjacent inputs $i,i^{{}^{\prime }}\in D$ and for an arbitrary subset of outputs O⊆K it holds [110]:

(4) $$\begin{array}{cc} & \;\text{Pr}\left[N\left(i\right)\in O\right]\leq e^{\epsilon }\text{Pr}\left[N\left(i^{{}^{\prime }}\right)\in O\right]+\delta \end{array}$$

For suitably small constants ϵ and δ, it is statistically impossible for an adversary to violate privacy because of the indistinguishability of neighboring datasets l and $l^{{}^{\prime }}$.

#### 3.3.1 DP with artificial noise

The preservation of privacy in OTA-FL can be achieved through the introduction of artificial noise to the local updates [111]. For instance, in [89], the authors consider OTA-FL with local DP over flat-fading Gaussian channels, where privacy-constrained artificial Gaussian noise is linearly combined with the local gradients during transmission. Analytical results show a tradeoff between privacy and convergence for certain loss functions, indicating that the training error decreases as the device count K increases. It is also demonstrated that the privacy level per user decreases at a rate of $O(1/\sqrt{K})$ compared to orthogonal transmission, where privacy leakage remains constant. However, a limitation of this scheme is that the device constrains the system signal-to-noise ratio (SNR) with the weakest channel, which can degrade learning accuracy. To address this issue, the MPA-DPFL scheme with misaligned power allocation is proposed by Yan et al. [92]. The scheme suggests allocating power misaligned when a device’s channel gain falls below a certain threshold, as opposed to the aligned manner used in [89].

Several works have considered more comprehensive approaches to introducing additional noise to local updates. Liao et al. [93], propose a novel DP-based method that adds spatially correlated perturbations to the local updates at each device. Compared to traditional DP-based methods that employ uncorrelated noise, this approach achieves higher learning accuracy while preserving defense ability. Additionally, for Hasircioǧlu et al. [94], it is emphasized that CSI at the devices is crucial for transmission and ensuring a DP-based privacy guarantee. The proposed distributed noise generation process is resilient against pilot attacks manipulated by a malicious server and the failure of transmitting nodes.

Another technique, presented by Elgabli et al. [95], is a so-called A-FADMM algorithm based on the alternating direction method of multipliers (ADMM). By adding a random variable to the local update and multiplying it with a random fading gain, this method preserves the privacy of both the local model trajectory and gradient trajectory. To enhance communication efficiency, Sonee et al. [96] propose the differentially private random projection FedSGD scheme, which reduces the dimension of local updates while preserving privacy.

#### 3.3.2 DP with channel noise

An alternative to artificial noise is that the inherent channel noise can also be leveraged to preserve privacy. Liu et al. [90] demonstrate that privacy can be obtained “for free” from the channel noise when the privacy constraint level is below a certain threshold that decreases with SNR. It is also highlighted that actively assigning additional power to perturb local gradients is generally suboptimal in OTA-FL scenarios. To address this, a dynamic power control strategy is optimized under power and privacy constraints to minimize the optimality gap. Similarly, in [91], the inherent receiver noise is harnessed to preserve DP against inference attacks, and a novel power control strategy is introduced. The analysis shows that the received SNR is primarily influenced by the number of devices when aiming for a higher level of privacy.

Adding artificial noise offers excellent control and allows the clients (or agents) to explicitly achieve the required privacy levels, not only at the BS but at any potential eavesdroppers as well. In the context of OTA-FL, artificial noise is expected to be added in the analog domain. This, unfortunately, would increase the hardware requirements for a wider dynamic range and better linearity of the RF chains, e.g., power amplifiers, at the transmitters, i.e., the clients.

Compared to adding artificial noise, using channel or receiver noise for DP contributes to energy saving of the clients. This is due to the fact that the transmit powers of the clients need to be low enough so that the receiver noise of the BS can successfully obfuscate the received local models at the BS. To this end, the transmit powers of the clients need to be carefully controlled to strike a delicate balance between signal reception and privacy. Moreover, the use of channel or receiver channel noise relies on truthful feedback from the BS on its noise level and channel conditions (from the clients). Even when the BS is trusted, it may not defend against eavesdropping attacks launched by unnoticed eavesdroppers located much closer to the clients than the BS [112]. To this end, the use of channel or receiver channel noise for DP may need to be implemented in coupling with cryptographic encryption (between the clients and the BS).

#### 3.3.3 Integration of DP and secure aggregation

Recent research has been focused on the joint design of secure and private OTA-FL systems. Xue et al. [97] consider a scenario where an external attacker equipped with a directional antenna eavesdrops on RF signals from the devices, which poses a more potent attack model that existing approaches struggle to handle. To address this challenge, the authors propose the introduction of pairwise suppressible random, artificial noise. These noises are used to obfuscate private local model parameters and thwart external eavesdroppers. This design can be seen as an integration of DP and secure aggregation at the physical layer for OTA-FL. In addition, Yan et al. [98] propose a secure and private OTA-FL framework, which utilizes noise to preserve privacy and security guarantees. The framework employs DP and MSE-security as the metrics. Specifically, a subset of devices is designated to send Gaussian artificial noise with the aim of degrading the SNR of potential eavesdroppers. To mitigate the impact of noise on learning accuracy, a channel-weighted post-processing mechanism is introduced. Moreover, the authors propose a scheduling algorithm based on the branch-and-bound concept with low complexity. This algorithm ensures the security of the system and the privacy of user data stored on the server.

## 4 Lessons learned, open challenges, and future directions

As OTA-FL in wireless environments continues to gain attention, researchers are actively working to tackle the associated challenges and improve system performance. However, there are still several open questions and directions for further research in this area. Some of these challenging questions are discussed in the following.

### 4.1 Aggregation distortion

OTA-FL systems face the challenge of distortion introduced by channel fading, noise, and transceiver filtering, which can degrade the quality of the received summation signal [113], [114]. Minimizing this distortion has been a persistent challenge in OTA-FL. Recently, coded OTA computation has been proposed for WFL. Zhu et al. [20] utilize one-bit quantization at the transmitter side to achieve one-bit OTA aggregation based on the signSGD algorithm [115]. The aggregated signal symbols are estimated through a majority voting-based decoder at the BS. Such a method combats the adverse effects of noise within the quantization error tolerance. Razavikia et al. [116] propose an OTA principle named ChannelComp to allow digital modulation to compute functions over MAC. ChannelComp discretizes the local models at the transmitters and requires a tabular mapping at the BS to determine the OTA-aggregated global models on the constellation map.

Indeed, coded OTA-FL can help reduce model distortions, offer compatibility with existing typically digital communication systems, and have the great potential to be integrated into existing systems. However, it adds complexity to the system with coding and decoding processes. Moreover, it could be non-trivial to extend coded OTA-FL to higher-order modulations and multi-antenna systems. On the other hand, uncoded OTA-FL requires an advanced transceiver design and delicate power control to achieve the optimal amplitude alignment and combat interference. Both coded and uncoded OTA-FL approaches necessitate improved design techniques to enhance the training performance of OTA-FL systems.

### 4.2 Stringent synchronization requirement

The assumption of perfect signal synchronization at the receiving end has been commonly made in most existing OTA-FL studies [19]. However, this assumption becomes increasingly challenging to achieve in scenarios with large system sizes and high heterogeneity. To achieve time synchronization in the uplink, some existing studies, such as [12] and [14], suggest that the existing synchronization techniques are reasonable for general OTA-FL systems, including timing advance used in 4G LTE and 5G NR (which controls device uplink transmission timing [117]). A method named AirShare is proposed by Abari et al. [118] to tackle the carrier frequency offset problems by enabling devices to share their broadcast clocks to implement frequency synchronization. Although these methods offer possible ways for OTA-FL implementation in practical systems, achieving precise synchronization in large-scale wireless systems is still a challenge and deserves further investigation. Goldenbaum et al. [119] also made efforts to address this challenge through robust designs. However, effectively implementing synchronization in complex and large-scale network environments remains an important and unresolved area that requires further investigation. Overcoming the synchronization challenge is crucial to ensure the reliable and efficient operation of OTA-FL in real-world wireless systems.

### 4.3 Data heterogeneity

In the domain of traditional FL, researchers have primarily focused on addressing the impact of data heterogeneity on model convergence. For instance, solutions like FedProx, introduced by Li et al. [120] have been devised to enhance training performance in the presence of heterogeneous networks by incorporating a proximal term on the client side. For Karimireddy et al. [121] Scaffold is developed to reduce data heterogeneity by implementing variance reduction-like techniques at the client end, thereby narrowing the gap with the global model.

Li et al. [122] define several non-I.I.D. distribution policies to serve as benchmarks. Meanwhile, the severely unbalanced distribution of data often leads to the gradient importance of different users, which makes some users’ updates submerged in receiver noise. An adequate design of aggregation weights under non-I.I.D. distributions is a vital direction in the future. However, all these algorithms are formulated under the assumption of ideal communication conditions and are only suitable for traditional FL.

Data heterogeneity poses a more pronounced challenge in the context of OTA-FL due to the analog nature of OTA aggregation. Considerable heterogeneity, such as variations in the sizes of local datasets. Consequently, the aggregation coefficients assigned to local models can lead to substantial discrepancies in the power levels of signals received by the BS from different clients. In this scenario, the resolutions of local models weighted by smaller aggregation coefficients significantly deteriorate, especially in the presence of non-negligible receiver noise. A near-far effect could occur, leading to the local models trained based on smaller amounts of data and delivered with lower transmit powers unaccounted for.

### 4.4 Secure and trustworthy OTA-FL

While efforts have been made to protect user data by submerging it within the overlay signal and mitigating the risk of information theft, there remains an inherent risk of leakage [123]. To ensure reliable aggregation computation and minimize aggregation errors caused by active eavesdroppers in different environments, it is crucial to design robust transceiver policies. These policies should effectively counteract the presence of eavesdroppers and maintain the integrity and privacy of the aggregated data. In addition to addressing security concerns, building users’ confidence in local data aggregation is paramount. To achieve this, it is essential to develop reliable algorithms and establish appropriate metrics in trustworthy FL. Researchers should focus their attention on creating robust and verifiable algorithms that instill trust in the aggregation process and provide transparent metrics to assess the reliability and accuracy of the aggregated results. By emphasizing the importance of trustworthy FL and dedicating efforts to its development, researchers can contribute to enhancing users’ confidence in the integrity and privacy of their data.

### 4.5 Other challenges

In addition, it is crucial to consider the impact of inherent channel fading and additive noise within OTA-FL systems on the convergence upper bound. These factors play a significant role in system performance and should be appropriately captured by a communication-learning metric. Identifying and defining a metric that effectively captures the influence of channel fading and noise is an essential direction for further research in OTA-FL. Moreover, the implementation of the right to data erasure within the OTA-FL framework presents challenges. The right to data erasure allows users to request the removal of their data from a dataset or model under specific circumstances to protect their privacy [124]. However, handling this “unlearning” situation in OTA-FL is not straightforward. Permanently removing user data from the system can lead to significant performance losses, particularly in non-I.I.D. data distributions. Therefore, developing techniques that address data erasure while minimizing the impact on system performance is a challenging aspect that requires further exploration in OTA-FL research.

## 5 Conclusion

This paper has provided a comprehensive overview of the latest studies on the emerging OTA-FL technique. We first categorized OTA-FL systems under different system settings, including single-antenna and multiple-antenna OTA-FL systems, as well as the consideration of RISs. The design objectives and optimization tools were analyzed. Next, we delineated the trust, security, and privacy aspects of OTA-FL systems, provided corresponding performance evaluation metrics, and unveiled critical concerns needed to promote better system design. Additionally, we highlighted the challenges faced by OTA-FL and suggested future research directions. Challenges to be holistically addressed include model distortion under channel fading, the ineffective OTA aggregation of local models trained on substantially unbalanced data, and the limited accessibility and verifiability of individual local models.

## CRediT authorship contribution statement

**Bingnan Xiao:** Writing – original draft, Methodology, Investigation, Conceptualization. **Xichen Yu:** Writing – original draft, Methodology, Investigation, Conceptualization. **Wei Ni:** Writing – review & editing, Supervision, Methodology, Conceptualization. **Xin Wang:** Writing – review & editing, Supervision, Methodology, Investigation, Funding acquisition, Conceptualization. **H. Vincent Poor:** Conceptualization, Writing – review & editing.
